# MiR-299-5p regulates apoptosis through autophagy in neurons and ameliorates cognitive capacity in APPswe/PS1dE9 mice

**DOI:** 10.1038/srep24566

**Published:** 2016-04-15

**Authors:** Yueqi Zhang, Chengeng Liu, Jinling Wang, Qiliang Li, Hong Ping, Shichao Gao, Peichang Wang

**Affiliations:** 1 Clinical Laboratory of Xuanwu Hospital, Capital Medical University, Beijing 100053, P.R. China; 2 Department of Medical Laboratory of Beijing Children’s Hospital, Capital Medical University, Beijing 100053, P.R. China

## Abstract

Abnormalities of autophagy can result in neurodegenerative disorders such as Alzheimer’s disease (AD). Nevertheless, the regulatory mechanisms of autophagy in AD are not well understood. Here, we describe our findings that microRNA (miR)-299-5p functions as an autophagy inhibitor by suppressing Atg5 and antagonizing caspase-dependent apoptosis. We observed decreased levels of miR-299-5p both in primary neurons under conditions of starvation and in hippocampi of APPswe/PS1dE9 mice. Additionally, low levels of miR-299-5p were observed in cerebrospinal fluid of AD patients. MiR-299-5p treatment resulted in attenuation of Atg5 and autophagy in primary neurons from APPswe/PS1dE9 mice, N2a cells and SH-SY5Y cells, whereas antagomiR-299-5p enhanced autophagy. Atg5 was verified as a direct target of miR-299-5p by dual luciferase reporter assays. Furthermore, transfection of miR-299-5p into primary hippocampal neurons caused the attenuation of caspase-mediated apoptosis, which was reversed upon starvation-induced autophagy. Inhibition of autophagy by shRNA knockdown of LC3β reduced apoptotic neuron death induced by antagomiR-299-5p. Injection of agomiR-299-5p into the cerebral ventricles of AD mice inhibited both autophagy and apoptosis and also improved the cognitive performance of mice. Overall, our results suggest that miR-299-5p modulates neuron survival programs by regulating autophagy. Thus, miR-299-5p serves as a potential neuroprotective factor in AD.

Alzheimer’s disease (AD) is a common clinical neurodegenerative disease characterized by the extracellular deposition of amyloid beta (Aβ) protein, the intracellular aggregation of Tau protein and the progressive loss of neuronal cells as the main pathological features. The pathogenesis of AD includes a variety of processes, such as oxidative stress, inflammatory damage, synaptic impairments and autophagy dysfunction^1^,^2^.

Autophagy is a catabolic process that is responsible for the clearance of long-lived proteins, organelles and protein aggregates^3^. Macroautophagy (hereafter referred to as autophagy) is a major kind of autophagy associated with extensive rearrangements of intracellular membranes. During the autophagy procedure, LC3I, as the cytoplasmic protein form of LC3, is processed and recruited to autophagosomes, where LC3II is generated by site-specific proteolysis and lipidation near the C-terminus. Thus, the formation of punctate cellular autophagosomes (autophagic vacuoles [AVs]) containing LC3II is a marker of autophagic activation. p62, interacting with LC3 and ubiquitin, is a selective autophagic substrate, and the first identified cargo receptor for autophagic degradation of ubiquitinated targets^4^. AVs are uncommon in brains devoid of AD pathology, but are particularly abundant in AD brains, suggesting that autophagy may be induced in AD^5^,^6^. The accumulation of AVs in affected neurons is responsible for Aβ production^7^. In young (4- to 6-month-old) AD mouse hippocampi, increased protein levels of the autophagosome marker LC3 are observed, demonstrating the induction of autophagy. Therefore, a delicate balance of autophagy maintains neuronal homeostasis, whereas an imbalance leads to the progression of neurodegeneration^8^.

MicroRNAs (miRs) are a class of endogenously expressed small noncoding RNA molecules in eukaryotes. By base-pairing with the 3′-untranslated regions (UTRs) of target mRNAs, miRs mediate posttranscriptional gene silencing^9^,^10^. The homeostasis of expression of miRs in the nervous system plays an important role in preventing neurodegeneration^11^. However, increasing evidence has shown that miRs are strongly associated with AD^12^. Because they are transported by liposomes or lipoproteins to prevent them from degradation, miRs can be stable in cerebrospinal fluid (CSF) and blood^13^. Therefore, several studies have been done to establish the potential role of miRs as biomarkers for differential diagnosis and disease progression monitoring^14^. As miRs have been shown to regulate many critical proteins mediating AD, miR-mediated regulation represents a new strategy with significant therapeutic prospects^15^.

In our previous studies, we identified specific miRs that can be used as biomarkers to diagnose AD^16^,^17^,^18^, and using microarray analysis, we showed that the expression of multiple miRs is significantly changed in the hippocampi of 9-months-old APP/PS1 double transgenic mice^16^. We hypothesized that among these changed miRs, some may regulate autophagy in AD. In this study, we show that miR-299-5p regulates both neuron autophagy and apoptosis *in vitro*. Moreover, exogenous expression of pharmacologic miR-299-5p by agomiR injection effectively reduces the apoptosis of hippocampi neurons and improves the cognitive capability of transgenic mice, demonstrating a possible therapeutic approach for AD.

## Results

### MiR-299-5p is dysregulated in AD and is associated with autophagy

To identify miRs that regulate autophagy in AD, we examined primary hippocampal neurons from APPswe/PS1dE9 AD model mice. We chose this model because APPswe/PS1dE9 mice show increased Aβ42 expression and impaired memory at 9 months^19^. Our study shows that the protein levels of LC3βII and Beclin1 are enhanced by 4 h culture of primary hippocampal neurons from APPswe/PS1dE9 mice in nutrient-deprived EBSS medium, and the protein levels of p62 are reduced (Supplementary Fig. S1), indicating a high autophagy level.

**Figure 1:**
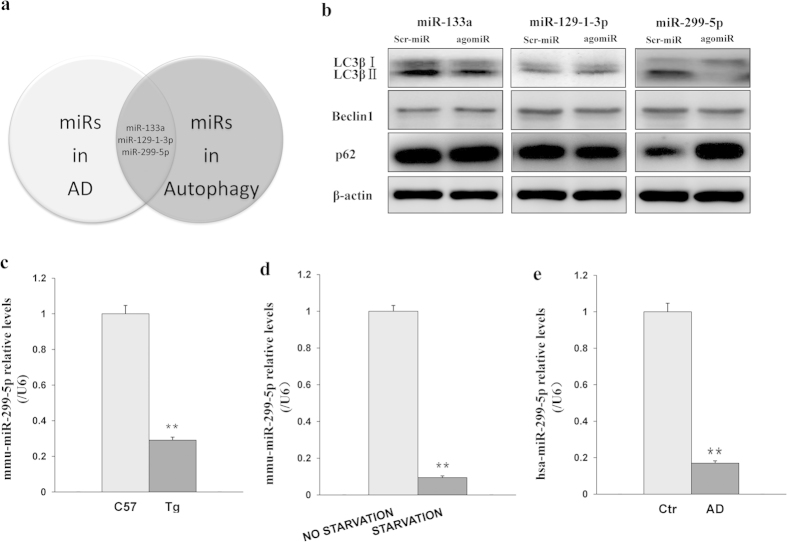
Decreased levels of miR-299-5p in autophagy and AD. (**a**) Venn diagram showing the AD-associated miRs (light) and the miRs that were altered under starvation conditions (dark). The overlapping 3 miRs were considered as potential regulators both in autophagy and in AD. (**b**) The 3 miRs or scrambled miRs (Scr-miR) were transfected into primary neurons for 24 h, and then autophagy related proteins were analyzed by western blotting (n = 3). β-actin was used as a loading control. (**c**) RT-PCR confirmed that mmu-miR-299-5p levels were lower in 9-month-old APPswe/PS1dE9 mice (Tg) than C57 mice (n = 4 per group). SnRNA U6 was used as an internal control. (**d**) RT-PCR analysis of endogenous miR-299-5p levels under no starvation or starvation (4 h) conditions. **P < 0.01. Bars indicated mean ± SD of 3 independent experiments. (**e**) RT-PCR showed downregulation of miR-299-5p in CSF of AD patients (n = 6 per group). **P < 0.01.

To identify miRs that regulate autophagy in APPswe/PS1dE9 hippocampal neurons, we used microarray analysis to compare miR levels for neurons cultured with and without FBS supplementation (Supplementary Table S1). The expression of miR-129-1-3p, miR-133a and miR-299-5p was markedly changed (>2-fold) both under conditions of nutrient starvation and in an AD-related microarray expression profiles^16^,^17^,^18^ (Fig. 1a). Of these 3 miRs, only miR-299-5p downregulated the protein levels of LC3βII and upregulated the expression of p62 in primary hippocampal neurons from APPswe/PS1dE9 mice, indicating significant inhibition of autophagy (Fig. 1b and Supplementary Fig. S2). To validate the microarray results, we performed RT-PCR in duplicate. Our analysis verified that miR-299-5p is downregulated in the hippocampi of 9-month-old APPswe/PS1dE9 mice (Transgenic mice, Tg) and under starvation conditions (Fig. 1c,d).

To determine whether, as for APPswe/PS1dE9 AD model mice, miR-299-5p is downregulated for AD patients, we assessed the level of miR-299-5p in human CSF from 6 AD patients and from 6 non-AD controls with a similar range of age, sex and educational status (Supplementary Table S2). In agreement with the mouse data, miR-299-5p was significantly downregulated in AD patients compared to the healthy controls (Fig. 1e).

### Atg5 is an autophagy-related target of miR-299-5p

**Figure 2:**
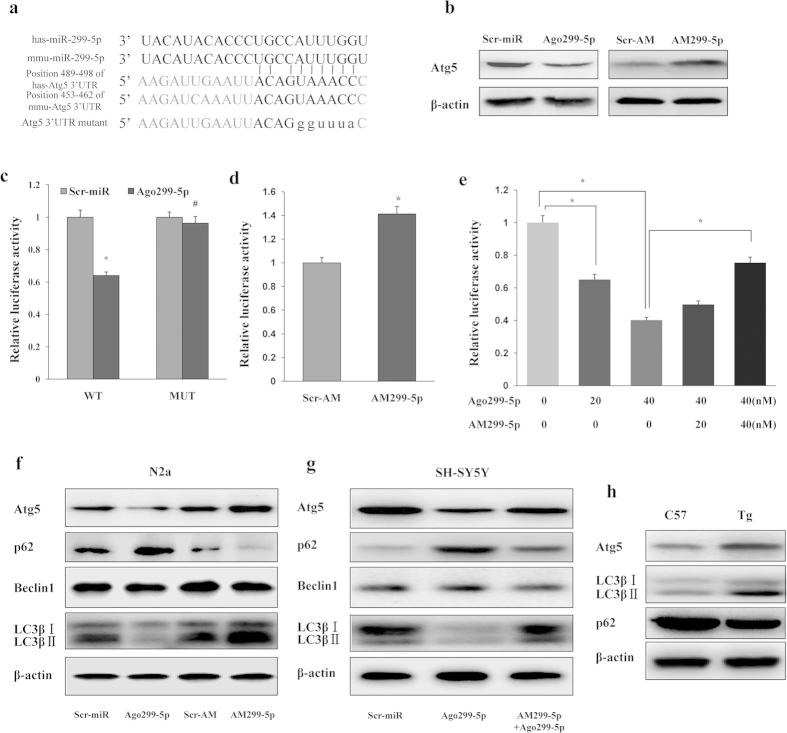
MiR-299-5p affects Atg5 levels directly and alters the levels of other autophagy-related proteins. (**a**) Conservation of miR-299-5p and the predicted binding sequences between miR-299-5p and target sites in the Atg5 3′UTR. Matches within the seed sequence are indicated. The mutated sequences are showed as lower case letters. (**b**) Primary hippocampal neurons from AD mice were transfected with agomiR-299-5p (Ago299-5p) or antagomiR-299-5p (AM299-5p). The levels of Atg5 protein were analyzed by western blotting. (**c**) Luciferase vectors were generated by inserting the wild-type (WT) or mutated (MUT) 3′UTR fragments of Atg5 position 489–498 into pmirGLO plasmid. Normalized luciferase activity in lysates from Hela cells were assayed 24 h after transfection (mean ± SD of independent experiments, n = 3, *p < 0.05. ^#^represents no significance). (**d**) AM299-5p increased luciferase activity when co-transfected with vectors containing a WT fragment (mean ± SD of independent experiments, n = 3, *p < 0.05). (**e**) Transfection with Ago299-5p decreased the luciferase activity of vectors containing WT fragment in a dose-dependent manner, and co-transfection with AM299-5p dose-dependently counteracted the effects of Ago299-5p in a dose-dependent manner (mean ± SD of independent experiments, n = 3, *p < 0.05). (**f**) In Neuro-2a (N2a) cells, Atg5 protein levels were reduced by transfection with Ago299-5p and increased by transfection with AM299-5p. Other autophagy-related proteins (LC3β and p62) were also modulated (n = 3). (**g**) In SH-SY5Y cells, transfection with Ago299-5p (40 nM) modulated Atg5, LC3β and p62 protein levels, and the effects were attenuated by co-transfection with AM299-5p (40 nM, n = 3). (**h**) Atg5 and LC3β protein levels in hippocampi of 9-month-old APPswe/PS1dE9 mice (Tg) and C57 mice (n = 4 per group).

To unravel the mechanisms of autophagy inhibition by miR-299-5p, we searched for autophagy-related targets. Atg5, an essential autophagy gene was identified as a putative target by TargetScan^20^ and miRBase^21^. The miR-299-5p targeting sequence of the human miR (hsa-miR-299-5p) is identical to that of its mouse homolog (mmu-miR299-5p) and is predicted to target Atg5 at one binding site within its 3′UTR (Fig. 2a). To determine whether miR-299-5p regulates Atg5 expression, we examined the protein levels of Atg5 in primary hippocampal neurons from APPswe/PS1dE9 mice after treatment with agomiR-299-5p (Ago299-5p) or antagomiR-299-5p (AM299-5p). Western blot analysis demonstrated that Ago299-5p attenuates the protein levels of Atg5, while AM299-5p increases Atg5 protein levels in transfected neurons (Fig. 2b and Supplementary Fig. S3a). To further verify that miR-299-5p directly targets Atg5 within its 3′UTR, we co-transfected either Scr-miR or Ago299-5p with luciferase vectors bearing WT or MUT target sequences from the predicted site at position 489–498. Ago299-5p reduced the luciferase activity in Hela cells co-transfected with a vector containing WT sequence, but not MUT sequence (Fig. 2c). Conversely, the luciferase activity of cells transfected with the WT sequence vector was enhanced by co-transfection with AM299-5p, but not its scrambled control (Fig. 2d). Furthermore, the effect of Ago299-5p in decreasing the luciferase activity of the WT vector was dose-dependent and was antagonized by AM299-5p in a dose-dependent manner (Fig. 2e). These results confirm that mAtg5 is a direct target of miR-299-5p.

To verify these findings in relevant neuronal cells, we transfected Neuro-2a and SH-SY5Y cells. Similar to the above results, Ago299-5p reduced the level of Atg5 protein in Neuro-2a cells, while AM299-5p caused the opposite effects (Fig. 2f and Supplementary Fig. S3b). In SH-SY5Y cells, miR-299-5p also decreased Atg5 expression, and this effect was partially antagonized by AM299-5p (Fig. 2g and Supplementary Fig. S3c). Nonetheless, the transfection with AM299-5p in SH-SY5Y cells did not increase the levels of Atg5 protein significantly (Supplementary Fig. S4a), potentially due to the low basal expression of miR-299-5p in SH-SY5Y cells (Supplementary Fig. S4b). Corresponding modulation of other autophagic proteins in Neuro-2a and SH-SY5Y cells is consistent with the physiological effects of miR-299-5p in regulating the autophagic pathway in these cells (Fig. 2f, g and Supplementary Fig. S3b,c). The levels of Atg5 protein were also increased in the hippocampi of 9-month-old APPswe/PS1dE9 (Tg) mice compared with the wild type control (C57BL/6) mice, with corresponding changes in LC3βII and p62 (Fig. 2h and Supplementary Fig. S3d).

### Effect of miR-299-5p on the cellular autophagy level

**Figure 3:**
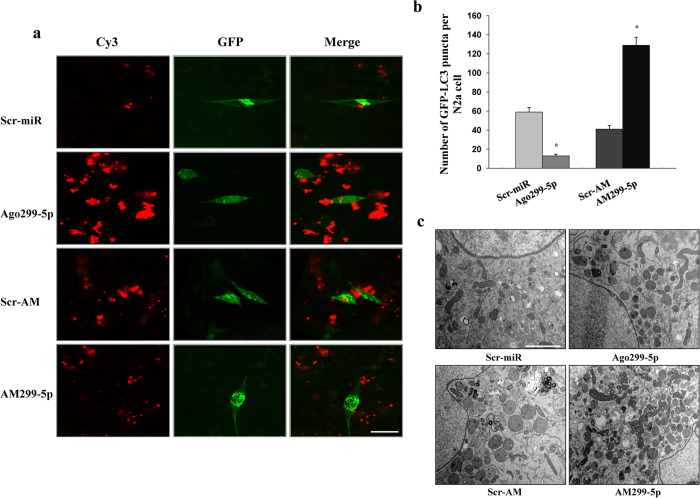
Assessment of autophagy in miR-299-5p (Ago299-5p) or antagomiR-299-5p (AM299-5p)-overexpressing cells. (**a**) Cy3-labeled Ago299-5p blocked GFP-LC3 puncta formation, and AM299-5p promoted puncta formation in Neuro-2a cells (n = 100). Scale bar = 25 μm. (**b**) Quantitative analysis of the experiments in panel (a) (mean ± SD of independent experiments, n = 4, *p < 0.05). (**c**) Representative TEM images of AVs in control and miR-299-5p/AM299-5p-overexpressing primary hippocampal neurons from AD mice. Scale bar = 2 μm.

To further explore the role of miR-299-5p in autophagy, we performed a GFP-LC3 puncta formation assay. In this assay, GFP-LC3 puncta as autophagy markers in the cytoplasm reflect the recruitment of LC3 proteins to autophagosomes. Cy3-labeled Ago299-5p or AM299-5p was cotransfected with GFP-LC3 into Neuro-2a cells. Scrambled miR (Scr-miR) or antagomiR (Scr-AM) served as negative controls (NC). There was a significant decrease of GFP-LC3 puncta in Ago299-5p-transfected cells. Furthermore, cells transfected with AM299-5p revealed a statistically significant increase in the autophagic flux distribution (Fig. 3a,b). These results suggest that miR-299-5p suppresses autophagosome accumulation.

We also examined the effects of transfecting Ago299-5 and AM299-5p into primary hippocampal neurons from APPswe/PS1dE9 mice. Ago299-5p was transfected to primary hippocampal neurons with high efficiency (Supplementary Fig. S5). We noted that the basal autophagy level was high with no nutrient limitation (serum and glucose deprivation) in primary hippocampal neurons from APPswe/PS1dE9 mice compared with C57 mice (Supplementary Fig. S6). However, 24 h after transfection of Ago299-5p into hippocampal neurons from APPswe/PS1dE9 mice, Atg5 protein levels were obviously decreased (Supplementary Fig. S7). We used transmission electron microscopy (TEM) scanning to determine whether Ago299-5p also effects autophagy in these cells. Our results show that miR-299-5p causes a significant decrease in AV formation in primary hippocampal neurons after 24 h transfection, while AM299-5p induces a significant increase in AVs (Fig. 3c). These results verify the role of miR-299-5p in primary neurons.

### MiR-299-5p ameliorates autophagy-related apoptosis in primary hippocampal neurons from AD mice

**Figure 4:**
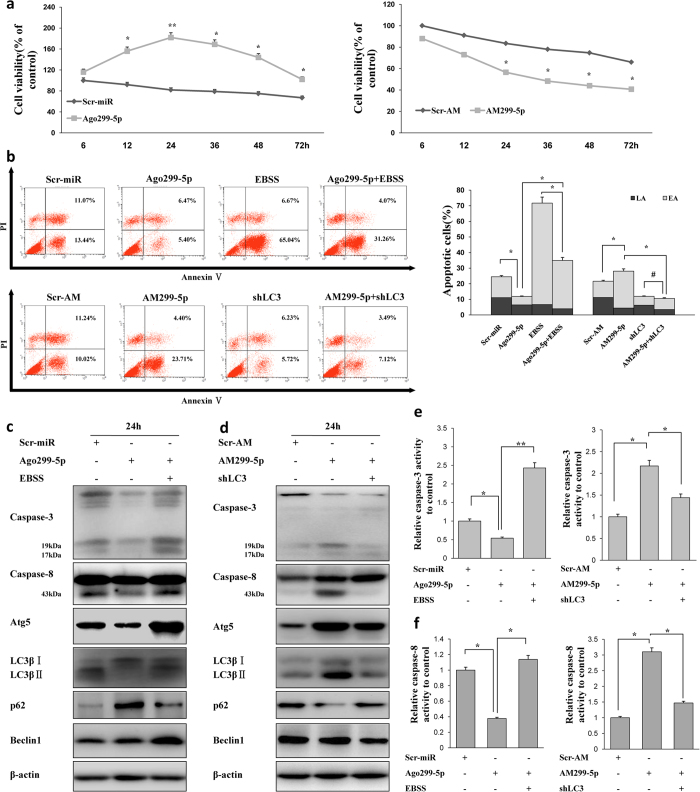
MiR-299-5p regulates autophagy and apoptosis in primary hippocampal neurons. (**a**) Primary hippocampal neurons were treated with Ago299-5p/AM299-5p or their scrambled controls at 40 μM for the indicated time and then cell viability was measured by MTT assay (mean ± SD of independent experiments, n = 3, *p < 0.05, **p < 0.01). (**b**) Top: EBSS culture accelerated apoptosis of Ago299-5p rescued neurons; Bottom: LC3β knockdown attenuated AM299-5p-induced apoptotic cell death. Primary neurons were pretreated for 20 h with Ago299-5p then cultured with EBSS for 4 h and stained with FITC-conjugated Annexin V/PI for flow cytometry. Primary neurons with or without stable LC3β knock down were treated with AM299-5p for 24 h followed by Annexin V/PI staining and flow cytometry. Right: The bar graph indicates the percentage of apoptotic neurons. The Annexin V positive and PI negative (early apoptotic, EA; light bars) and Annexin V and PI double positive (late apoptotic, LA, dark bars) apoptotic neurons are shown (mean ± SD of independent experiments, n = 3, *p < 0.05, ^#^represents no significance). (**c**) Primary neurons were transfected with scrambled control or Ago299-5p for 24 h. or primary neurons were transfected with Ago299-5p for 20 h then switched to EBSS medium for an additional 4 h (totally 24 h). Western blot analysis was performed with antibodies against caspase-3, -8, Atg5, LC3β, p62 or Beclin1. β-actin was tested as loading control (n = 3). (**d**) Primary neurons with or without stable LC3β knockdown were treated with AM299-5p or its scrambled control for 24 h. Western blot analysis was performed using the indicated antibodies (n = 3). (**e**) Neurons were treated as in (**c**,**d**); caspase-3 activity was then measured using a commercial kit. Data are normalized to scrambled controls (mean ± SD of independent experiments, n = 3, *p < 0.05, **p < 0.01). (**f**) Neurons were treated as in (c and d); caspase-8 activity was then measured using a commercial kit. Data are normalized to scrambled controls (mean ± SD of independent experiments, n = 3, *p < 0.05).

To assess the effects of miR-299-5p on cell growth of primary hippocampal neurons from APPswe/PS1dE9 mice, primary neurons were transfected with Ago299-5p or AM299-5p and then evaluated by MTT assay. The results show that Ago299-5p promotes time-dependent cell proliferation, and conversely, AM299-5p inhibits proliferation (Fig. 4a). Furthermore, in an Annexin V/PI double staining assay, the percentage of AnnexinV-positive/PI-negative (early apoptotic; EA) cells was markedly increased after culture under nutrient-depleted conditions (EBSS), and transfection of Ago299-5p reversed this effect (Fig. 4b, top panel). On the other hand, knockdown of LC3 (Supplementary Fig. S8) led to a reduction in the percentage of EA cells, and transfection of AM299-5p reversed that effect (Fig. 4b, bottom panel). These results indicate that autophagy inhibition by miR-299-5p attenuates apoptosis in primary hippocampal neurons from AD mice.

To further reveal the mechanisms of inhibition of apoptosis in primary hippocampal neurons, the effect of miR-299-5p on the activation of apoptosis-related proteins was analyzed by western blotting. There were no distinct changes in Bax, Bcl-2 and caspase-9 protein levels (Supplementary Fig. S9); however, a significant upregulation of caspase-3 and caspase-8 cleavage levels was observed 24 h after treatment with 40 nM miR-299-5p. This up-regulation was accompanied by the modulation of autophagy-associated proteins and was reversed by culture in EBSS (Fig. 4c and Supplementary Fig. S10a). Conversely, AM299-5p treatment increased the cleaved forms of caspase-3 and caspase-8, and this increase was reversed by LC3 knockdown (Fig. 4d and Supplementary Fig. S10b). To further examine the effect of miR-299-5p on autophagy-associated apoptosis, caspase-3 and caspase-8 were analyzed using caspase activity kits. Consistent with the western blotting results, Ago299-5p inhibited caspase-3 and -8 activity, and this inhibition was reversed by culture in EBSS; the activity of caspase-3 and -8 was also enhanced by AM299-5p, and this enhancement was reversed by LC3 knockdown (Fig. 4e,f). Taken together, these results suggest that the inhibition of autophagy by miR-299-5p reduces apoptosis by suppressing the activation of caspase-3 and caspase-8 in primary hippocampal neurons from AD mice.

### MiR-299-5p inhibits both autophagy and apoptosis in APPswe/PS1dE9 mice

**Figure 5:**
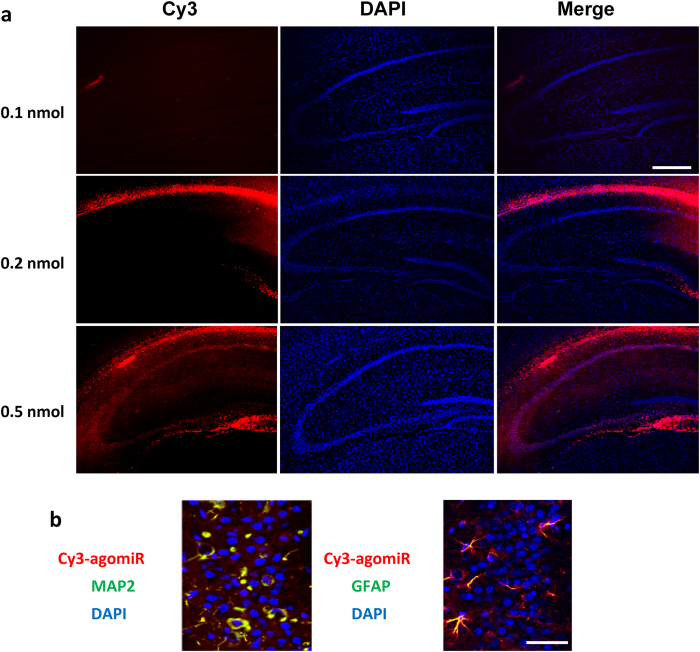
After injection, Cy3-labeled Ago299-5p is distributed throughout the brain tissues. (**a**) 9-month-old APPswe/PS1dE9 mice were injected with different doses of Cy3-labeled Ago299-5p into the third ventricle (n = 3). Fluorescence of Cy3 was disseminated throughout the hippocampi and surrounding tissues after injection with 0.5 nmol for 24 h. Scale bar = 250 μm. (**b**) Cy3-labeled Ago299-5p was absorbed into MAP2-positive neurons and GFAP-positive glial cells. Scale bar = 25 μm.

To evaluate the function of miR-299-5p *in vivo*, Cy3-labeled Ago299-5p was injected into the third ventricle of 9-month-old APPswe/PS1dE9 mice at different dosages. As shown in Fig. 5a, Cy3-labeled Ago299-5p was disseminated throughout the hippocampus and surrounding tissues after treatment with 0.5 nmol for 24 h. Moreover without transfection reagent, Ago299-5p was absorbed by MAP2-positive neuronal cells and glial fibrillary acidic protein (GFAP)-positive glial cells (Fig. 5b).

**Figure 6:**
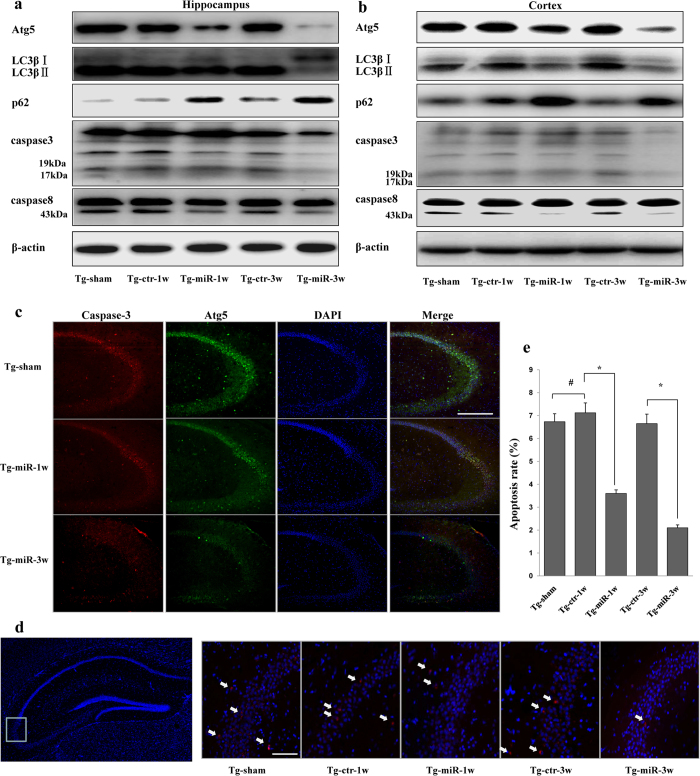
Therapeutic effects on protein levels. (**a**) The alteration of protein levels in the hippocampus after treatment with Ago299-5p (n = 5 per group). (**b**) The alteration of protein levels in cortex after treatment with Ago299-5p (n = 5 per group). (**c**) The fluorescence intensity of cleaved caspase-3 in the hippocampus was weakened with the decreasing intensity of Atg5 after treatment with Ago299-5p (n = 5 per group). Scale bar = 250 μm. (**d**) Apoptosis examination was performed by TUNEL assay, TUNEL positive cells were marked with arrows. Scale bar = 50 μm. Representative TUNEL assay images were captured from the boxed region. (**e**) The percentage of positive cells was calculated as the apoptosis rate (number of positive cells/total number of cells × 100%) (mean ± SD, n = 5 per group, *p < 0.05, ^#^represents no significance).

As Ago299-5p suppressed the autophagy and apoptosis of neurons *in vitro*, we assessed its effects on autophagy and apoptosis in the hippocampus of APPswe/PS1dE9 mice. At both 1 and 3 weeks after injection, the protein levels of Atg5 and caspase-8 were reduced and p62 was increased in mice treated with Ago299-5p compared with the control mice. Caspase-3 and LC3βII were also reduced at 3 weeks after treatment with Ago299-5p (Fig. 6a and Supplementary Fig. S11a). In the cortex of miR-299-5p treated mice, similar changes in protein levels were observed (Fig. 6b and Supplementary Fig. S11b). The decreases in Atg5 and cleaved caspase-3 protein in hippocampi were confirmed by immunofluorescence staining using an independent set of samples (Fig. 6c). In addition, the hippocampi of mice that were injected with Ago299-5p at 1 and 3 weeks had fewer TUNEL-positive nuclei than those of control mice (Fig. 6d,e), indicating that Ago299-5p suppresses cell apoptosis.

APPswe/PS1dE9 mice develop amyloid plaques formation starting around 4 months of age^22^. We investigated whether intracerebral injection of Ago299-5p can alter the level of Aβ42. Our results demonstrate that APPswe/PS1dE9 mice treated with Ago299-5p show a trend of reduction in Aβ42 formation in hippocampi and cortexes that is statistically insignificant (Supplementary Fig. S12a). Similarly, the level of Aβ42 from the hippocampal region showed no remarkable reduction in Ago299-5p treated mice as assessed by ELISA analysis (Supplementary Fig. S12b). These data suggest that miR-299-5p treatment had no significant effect on the levels of Aβ42 in APPswe/PS1dE9 mice.

### Intracerebral injection of miR-299-5p improves the cognitive performance of APPswe/PS1dE9 mice

**Figure 7:**
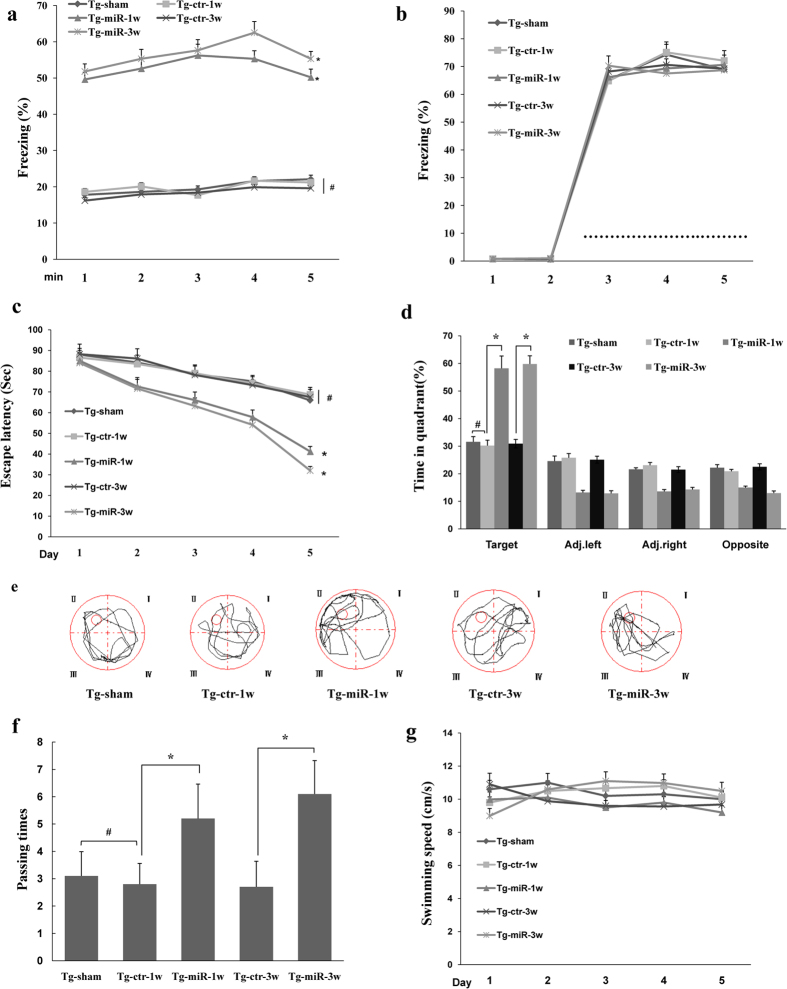
Behavior changes of APPswe/PS1dE9 mice after treatment with Ago299-5p. (**a**) In a contextual fear conditioning test, the freezing time for APPswe/PS1dE9 mice treated with Ago299-5p was higher than for Scr-miR-treated transgenic mice (Tg-ctr) or sham untreated mice (Tg-sham), both at 1 and 3 weeks after injection. (**b**) In the cued conditioning test, there was no significant difference between these five groups. The sound cue intervention is showed as dotted line. (**c**) In the MWM test, the escape latency to reach the hidden platform during the acquisition phase was determined over 5 days. (**d**) The percentage of time mice spent in the four quadrants during the probe trial of the MWM test. (**e**) Representative swimming trajectories in the probe trial. (**f**) Passing times in the probe trial. (**g**) The average swimming speed during the water maze training (mean ± SD, n = 5 per group, *p < 0.05 compared with control group, ^#^represents no significance).

To determine whether the injection of Ago299-5p can ameliorate the memory deficit in APPswe/PS1dE9 mice, we performed behavioral tests in 9-month-old mice (Supplementary Fig. S13). A contextual fear conditioning test, which can assess associative memory associated with the hippocampus^23^, was first performed. As shown in Fig. 7a, at both 1 and 3 weeks after injection, the freezing time for APPswe/PS1dE9 mice treated with Ago299-5p was higher than for mice treated with Scr-miR (Tg-ctr) or sham untreated mice (Tg-sham), demonstrating that miR-299-5p enhances hippocampal memory function. In a cued conditioning test in which the amygdala plays an important role, there was no difference between the five groups of mice (Fig. 7b). There was also no statistical difference in freezing response among groups when tested by fear conditioning training (data not shown), suggesting similar conditional fear learning ability. We also performed a Morris Water Maze (MWM) test and found that APPswe/PS1dE9 mice treated with Ago299-5p took significant less time than control mice to find a hidden platform at both 1 and 3 weeks after injection (Fig. 7c). Additionally, in a probe trial, APPswe/PS1dE9 mice treated with miR-299-5p at either 1 or 3 weeks spent a significant longer time than control mice in the target quadrant that formerly contained the platform (Fig. 7d), and the times of passing through the platform were also significantly increased (Fig. 7e,f). However, no significant difference in swimming speed was observed among the five groups during a probe trial (Fig. 7g). Collectively, these data indicate that treatment with Ago299-5p improves the spatial memory function of APPswe/PS1dE9 mice.

## Discussion

In this study, we demonstrate that miR-299-5p inhibits autophagy by downregulating Atg5, and that autophagy modulated by miR-299-5p is closely associated with caspase-mediated apoptosis in neurons. The results demonstrate for the first time that miR-dependent dysregulation of Atg5 takes part in the autophagy of AD and, furthermore, that miR-299-5p may potentially be beneficial to the treatment of this disease.

In the nervous system, autophagy plays an important role in regulating cellular homeostasis^24^. Progressive transcriptional down-regulation of autophagy occurs in the normal human brain upon aging. In addition, autophagy is up-regulated in the brains of AD patients versus normal aging brains^6^. In TgCRND8 mice, a model for AD, abundant abnormally enlarged autolysosomes are observed at 6- and 12-months of age^25^. For 9-months-old PS1/APP mice compared with age-matched non-transgenic mice, the numbers of AVs are at least 23-fold higher in neurons^26^. Despite emerging data indicating that the pathologic process in AD is closely related to autophagy, the upstream pathway of autophagy in AD is unclear. Because there is some correlation between miRNA and autophagy^27^,^28^,^29^, we postulated that miRs may regulate autophagy associated with the pathological process of AD. Through microarray and western blotting analyses, we identified miR-299-5p as a potent autophagy regulator.

MiR-299-5p is known to be involved in many pathophysiological processes, including prostate cancer^30^, myelopoiesis^31^ and primary biliary cirrhosis^32^. Nevertheless, the function of miR-299-5p in pathophysiological processes is likely to be determined by a wide variety of targets in a disease and tissue-specific manner^33^.

LC3βII, Beclin1, Atg5 and p62 play indispensable roles in autophagosome formation, with LC3βII being the most reliable biomarker of autophagy^34^. P62 is selectively incorporated into autophagosomes by directly binding to LC3 and is effectively degraded by autophagy. Thus, the total cellular levels of p62 inversely correlate with autophagic activity^35^.Beclin1 acts in the induction step prior to autophagy^36^ and constitutes PI3P, a signaling lipid required for the recruitment of autophagy effectors with class III phosphatidylinositol 3-kinase^37^. In this study, we used TEM scanning of AVs and other methods to demonstrate that miR-299-5p inhibits neuronal autophagy both *in vivo* and *vitro* as verified by the change of LC3βII, Beclin1, Atg5 and p62.

MiRs have the capability of simultaneously targeting a large number of genes for posttranscriptional repression through sequence-specific binding to sites within 3′UTRs^38^, indicating a multiple target effect that can cause changes in the activities of important cellular processes. To determine whether the anti-apoptotic effect of miR-299-5p on neurons is associated with the suppression of autophagy, we examined the effects of autophagy induction by EBSS and autophagy suppression by shLC3 on miR-299-5p function. Our results demonstrate that autophagy is closely associated with apoptosis, and additionally that the activation of caspase-3 and caspase-8 is triggered during the autophagy process. Caspase-3, as an apoptotic effector, is activated by caspase-8 cleavage^39^. In 6-month-old PS/APP mouse brains, immunoreactivity to activated caspase-3, as detected by immunogold TEM, accumulates in AVs when clearance is impaired^40^. The maturation of AVs to lysosomes is impaired during AD pathology, thereby impeding the elimination of AV contents^5^,^26^. To our knowledge, these results provide the first demonstration of caspase-8 cleavage in hippocampal neurons from APPswe/PS1dE9 mice during verified autophagy.

**Figure 8:**
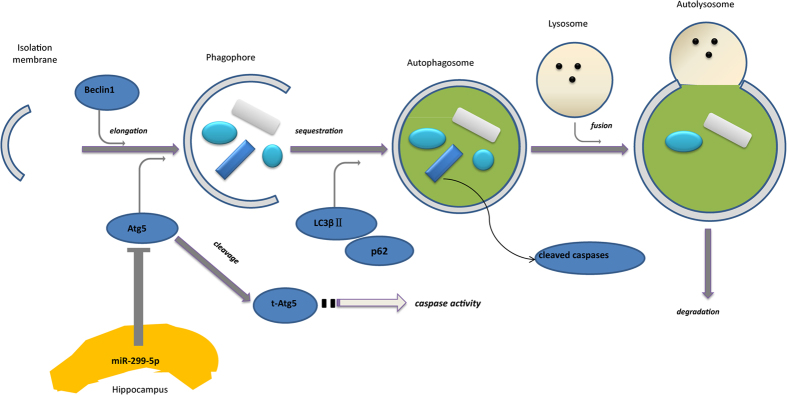
Model of miR-299-5p effect on autophagy and apoptosis in AD. MiR-299-5p suppresses autophagy by targeting Atg5, which functions at the stage of elongation of membranes. Atg5 induces caspase activation following cleavage of Atg5 by calpain. Cleaved caspase-3 accumulates in autophagosomes. In addition caspase-8 may be cleaved in a similar fashion.

The miR-299-5p target, Atg5, conjugates with Atg12 and Atg16 and then participates in autophagosome closure (sequestration step)^41^. Previous studies show that overexpression of Atg5 enhances susceptibility toward apoptotic stimuli irrespective of the cell type^42^. The calpain-cleavage product of Atg5 translocates to mitochondria and thereby triggers cell death involving cytochrome c and caspase-3 cleavage and release. There is also evidence that Atg5 interacts with Fas-associated protein with death domain (FADD) *in vitro* and *in vivo* and thereby triggers cell death involving caspases^43^. Taken together, Atg5 can regulate components of both the extrinsic and intrinsic apoptotic pathway. In the present study the downregulation of Atg5 by miR-299-5p may have potential impact on neuron survival (Fig. 8).

Despite the development of new therapeutic approaches for AD in the last decades, the morbidity rates still remain unacceptably high^44^. Thus, there is a great urgency to develop more efficient novel therapeutic approaches. The potential regulation of autophagy by miRs may offer an efficient multi-target strategy to accomplish robust autophagy inhibition. Further study should be performed to assess the side effects of miRs through long term treatment.

## Materials and Methods

### AD transgenic mice

APPswe/PS1dE9 mice were purchased from the Institute of Laboratory Animal Science, Chinese Academy of Medical Sciences & Comparative Medical Center. All animal experiments conformed to the National Institutes of Health guidelines. All animal protocols were approved by the ethics committee of Xuanwu Hospital of Capital Medical University.

### Cell culture and transfection

Primary neuronal cultures were prepared from hippocampi of embryonic day 16 fetuses of the APPswe/PS1dE9 mouse line. After mechanical dissociation, cells were cultured on plates and maintained in Neurobasal medium (Gibco, Invitrogen, USA) containing 2% B27 supplement (Gibco, Invitrogen, USA) and 100 units/ml penicillin/streptomycin (Gibco, Invitrogen, USA) at 37 °C in a humidified 5% CO_2_ atmosphere. After 24 h in culture, the cells were treated with 4 μg/mL cytarabine (Gibco, Invitrogen, USA) to prevent proliferation of non-neuronal cells. Follow-up experiments were performed after 7 days in culture. SH-SY5Y, N2a and Hela cells were maintained in DMEM + 10% FBS at 37 °C in a 5% CO_2_-humidified incubator. Primary neurons, N2a, SH-SY5Y or Hela cells were transiently transfected with Ago299-5p, AM299-5p or a nonspecific control (GenePharma, Shanghai, China) using Lipofectamine 2000 (Invitrogen, Carlsbad, CA, USA) according to the manufacturer’s instruction. Five samples per group were tested.

### miRNA microarray

For autophagy induction by starvation, primary hippocampal neurons were cultured in Earle’s balanced salt solution (Invitrogen, USA). After 6 h, cultured neurons were harvested for miR microarray analysis (miR microarray release, Agilent, USA).

### Protein analysis

Frozen hippocampi or cultured cells were homogenized in ice-cold RIPA buffer (Beyotime Biotechnology, Jiangsu, China) containing 0.5 mM PMSF. Insoluble material was removed by centrifuging the homogenates at 16 500 *g* for 15 min at 4 °C. The protein concentrations of the supernatants were quantified using a BCA protein assay kit (Beyotime Biotechnology, Jiangsu, China). Protein samples were boiled for 5 min with loading buffer, and proteins (30 μg per well) were separated by 15% SDS-polyacrylamide gel electrophoresis (Beyotime Biotechnology, Jiangsu, China) and then transferred onto nitrocellulose membranes (Millipore, Watford, UK). Membranes were blocked with 5% nonfat milk in PBST for 1 hour and then incubated at room temperature with PBST solution containing 3% BSA. Subsequently, the following primary antibodies were added: anti-Atg5 (Santa Cruz, CA, USA), anti-Beclin1 (Santa Cruz, CA, USA), anti-LC3β (Sigma-Aldrich St. Louis, USA), anti- p62 (Santa Cruz, CA, USA), anti-β-actin (Santa Cruz, CA, USA), anti-caspase-3,-8,-9 (Cell Signaling Technology, MA, USA), anti-bax (Santa Cruz, CA, USA), and anti-bcl-2 (Santa Cruz, CA, USA). Membranes were washed and incubated with horseradish peroxidase-conjugated anti-IgG antibody at room temperature for 1 hour. Bands were revealed by chemiluminescence, and band intensities were quantified using ImageJ software.

### Human CSF samples

The design of this study was approved by the ethics committee of Xuanwu Hospital of Capital Medical University, and all patients and controls or their legally authorized representatives provided individual informed consent. The study was conducted in accordance with the tenets of the Declaration of Helsinki. A total of 12 subjects were enrolled, including 6 patients with dementia of Alzheimer type (DAT) (mean age = 77.1 y, ranging from 72 to 82) and 6 age-matched normal controls (mean age = 76.1 y, ranging from 71 to 82). Diagnoses were made by doctors within the neurology department, Xuanwu Hospital of Capital Medical University. None of the patients had other malignancies or active pulmonary disease. A 5 ml volume of CSF was obtained by lumbar puncture, shipped on dry ice and stored in liquid nitrogen until analysis

### RNA extraction and real time PCR

Total RNA was isolated from individual CSF samples and harvested cells with an RNA extraction kit (Qiagen, Hilden, Germany). Stem-loop real time PCR for miR-299-5p was performed using a commercial kit (*GenePharma*, Shanghai, China) according to the manufacturer’s protocol. For Atg5 mRNA quantification, isolated RNA was reverse-transcribed using PrimeScript™ RT reagent (Takara, Shiga, Japan). Levels of β-actin mRNA were used as an internal standard to normalize the individual gene expression level of Atg5, and U6 snRNA as a standard for miR-299-5p. PCR primers were as follows: hsa-ATG5 forward, 5′-AGCAACTCTGGATGGGATTG-3′, reverse, 5′-CACTGCAGAGGTGTTTCCAA-3′; mmu-Atg5, forward, 5′-CTGGATGGGACTGCAGAATG-3′, reverse 5′-CGGAACAGCTTCTGGATGAA-3′; hsa-β-actin, forward, 5′-AGCGAGCATCCCCCAAAGTT-3′, reverse 5′-CGGAACAGCTTCTGGATGAA-3′; mmu-β-actin, forward, 5′-GTCCCTCACCCTCCCAAAAG-3′, reverse 5′-GCTGCCTCAACACCTCAACCC-3′; hsa/mmu-U6, forward, 5′-CGCTTCGGCAGCACATATAC-3′, reverse 5′-TTCACGAATTTGCGTGTCAT-3′. Quantitative real-time PCR was performed in the LightCycler 480 System (Roche, Mannheim, Germany) using the 2^−ΔΔCt^ method.

### Target prediction

Conventional online programs, including TargetScan^20^ and miRBase^21^ were used to predict the targets of miR-299-5p. The targets were further demonstrated and verified by the experiments described below.

### Construction of plasmid and luciferase assays

The 3′UTR of ATG5 was amplified by PCR and cloned into pmirGLO Dual-Luciferase miRNA target expression vector (Promega, Madison, WI).The predicted target site was mutated by site-directed mutagenesis. For luciferase reporter assays, wild type and mutated luciferase plasmid and agomiR/antagomiR were cotransfected into Hela cells. Luciferase activities were measured using the Dual-Glo^®^ Luciferase Assay System (Promega, Madison, WI, USA) on a Varioskan Flash microplate reader (Thermo Scientific, MA, USA) according to the manufacturer’s protocols. Firefly luciferase activity was normalized to Renilla luciferase activity for analysis. Each experiment was repeated in triplicate.

### Annexin V and PI staining

Cells were treated with the indicated concentrations of drugs for 24 h. Complete culture medium was replaced with EBSS solution for the EBSS group 4 h before harvest to induce autophagy. The apoptosis levels of neurons in the harvested cells (1 × 10^6^/ml) were assessed by staining with FITC-labeled Annexin V and PI (BD Pharmingen, San Diego, CA). In this assay, different labeling patterns enabled us to identify different cell populations: live cells (Annexin V-FITC negative/PI negative), early apoptotic cells (Annexin V-FITC positive/PI negative), late apoptotic/necrotic cells (Annexin V-FITC positive/PI positive) and dead cells (Annexin V-FITC negative/PI positive). Data was analyzed with a FACSCalibur flow cytometer (BD Biosciences).

### RNA interference

Stable depletion of LC3 was carried out by infection of neurons with shRNA-expressing lentiviral particles (Santa Cruz, CA, USA) and selection with puromycin (5 μg/ml). Positive clones were screened by western blotting. The neurons were allowed to proliferate for 1 week before any experimental procedures. Control shRNA lentiviral particles (Santa Cruz, CA, USA) were used for comparison.

### Cell viability

Neurons (2 × 10^3^) were plated in 96-well plates and incubated at 37 °C until the cells reached 30–40% confluence. The neurons were then treated with Ago299-5p, AM299-5p or a negative control at the concentration of 40 nM. Cell proliferation was assessed 24 h after treatment. MTT solution (20 μl at 5 mg/ml; Beyotime, Jiangsu, China) was added to each well, and after 4 h of incubation at 37 °C, the supernatant was discarded and 150 μl of DMSO were added. After 10 min of low speed shaking (100 rpm), the absorbance at 490 nm was read by a plate reader (Bio-Rad 680, CA, USA). All experiments were performed in biological triplicate.

### Caspase activity assay

Caspase-3/8 activity was determined using a caspase-3/8 activity assay kit (Beyotime, Haimen, China) according to the manufacturer’s instruction. Neurons were lysed after transfection with Ago299-5p, AM299-5p or a scrambled control (40 μM) for 24 h. The fluorescence was measured on 96-well plates using the Varioskan Flash microplate reader (Thermo Scientific, MA, USA). All experiments were performed in triplicate.

### Quantitative GFP-LC3 analysis

For GFP-LC3 analysis, 24 h after miR and GFP-LC3 cotransfection, GFP-LC3 puncta were quantitatively analyzed in each cell. Cells presenting a mostly diffuse distribution of GFP-LC3 in the cytoplasm and nucleus were considered as non-autophagic, and cells representing several intense punctate GFP-LC3 aggregates were considered as autophagic. GFP-LC3-positive or endogenous LC3-positive punctate structures were obtained by counting at least 100 positive cells in each working condition of 4 independent experiments. Fluorescence emission was detected using a confocal laser scanning microscope (Carl Zeiss, Jena, Germany).

### TEM scanning

Transmission electron microscopy was performed to assess autophagy of the primary neurons 24 h after treatment with agomiR or antagomiR. Fixed cells were post-fixed in 2% OsO_4_, dehydrated in graded alcohol and flat embedded in Epon 812 (Electron Microscopy Sciences, Fort Washington, PA, USA). Ultra-thin sections (100 nm) were prepared, stained with uranyl acetate and lead citrate, and examined by electron microscopy (Hitachi H7500, Japan).

### Microinjection of AD mice with agomiR

Animals were anesthetized with 4% chloral hydrate (10 ml/kg, i.p.) and positioned in a stereotaxic apparatus. Ago299-5p was labeled with Cy3 to track its diffusion. A total of 0.5 nmol of Ago299-5p or scrambled control (GenePharma, Shanghai, China) dissolved in 1 μl buffer was injected into the third ventricle at a rate of 0.2 μl/min. Frozen sections were prepared from treated mouse brains 24 h after injection. The fluorescence of Cy3 was observed under confocal laser scanning microscope (Carl Zeiss, Jena, Germany).

### Fear conditioning and MWM testing

Behavioral tests of mice were performed in a silent, isolated room at a range of constant temperatures (20–24 °C). Cue and contextual fear conditioning tests, which can assess hippocampal associated memory, were performed as described^45^. MWM testing was also carried out to measure spatial learning and memory performance of the mice^46^. Swimming trajectory was video-taped and analyzed (Institute of Zoology, Chinese Academy of Sciences, Beijing, China).

### Immunofluorescence

Frozen sections were air-dried at room temperature for 15 min and with 4% paraformaldehyde for 10 min. Then sections were rinsed with PBST (0.25% Triton-X100) and blocked for 30 min at room temperature in 5% goat serum. After incubation with primary antibodies diluted in PBS for 2 h at room temperature, sections were washed with PBS and incubated with secondary antibodies for 1 h at room temperature. Sections were washed again and mounted with mounting medium containing DAPI (Vector Lab, Burlingame, USA). Primary antibodies used were as follows: anti-cleaved caspase-3 (1:300, Cell Signaling Technology, MA, USA), anti-Atg5 (1:400, Santa Cruz, CA, USA), anti-MAP2 (1:400, Santa Cruz, CA, USA), anti-GFAP (1:300, Abcam, Cambridge, UK), and anti-Aβ42 antibody (Invitrogen, CA, USA). All immunofluorescence images were representative of at least three independent animal experiments.

### TUNEL staining

To detect apoptotic cell death, frozen embedded sections were stained by the TUNEL technique using an *in situ* apoptosis detection kit (Keygen Biotech, Nanjing, China) according to the protocol provided by the manufacturer. The localized red fluorescence of apoptotic cells was detected with a confocal laser scanning microscope (Carl Zeiss, Jena, Germany). The CA1, CA2 and CA3 of the hippocampus were scanned with high-power magnification. The apoptotic index was then calculated as follows: AI = (number of apoptotic cells per section/total number of cells per section) × 100%. Experiments were performed in triplicate.

### Data analysis and statistics

Statistical analysis was performed using GraphPad Prism software (version 5.01; California, USA). All data are presented as the mean ± standard deviation. Data were analyzed using one-way or two-way ANOVA (as appropriate) followed by a Bonferroni’s post hoc test. Further intergroup comparisons were also performed using a two-tailed t test. P-values less than 0.05 (*p* < 0.05) were considered significant.

## Additional Information

**How to cite this article**: Zhang, Y. *et al*. MiR-299-5p regulates apoptosis through autophagy in neurons and ameliorates cognitive capacity in APPswe/PS1dE9 mice. *Sci. Rep*. **6**, 24566; doi: 10.1038/srep24566 (2016).

## Supplementary Material

Supplementary Information
