# Supplementary material for: MiR-299-5p regulates apoptosis through autophagy in neurons and ameliorates cognitive capacity in APPswe/PS1dE9 mice
Source: Sci Rep. 2016 Apr 15;6:24566. doi: 10.1038/srep24566 (PMC4832239; doi:10.1038/srep24566)
Supplement: Supplementary Information [file srep24566-s1.doc]

**SUPPLEMENTARY INFORMATION**

1. Supplementary Table S1-S2
2. Supplementary Figure S1-S13

**MiR-299-5p regulates apoptosis through autophagy in neurons and ameliorates cognitive capacity in APPswe/PS1dE9 mice**

Yueqi Zhang^1^, Chengeng Liu^1^, Jinling Wang^1^, Qiliang Li^2^, Hong Ping^1^, Shichao Gao^1^, Peichang Wang^1,*^

^1^Clinical Laboratory of Xuanwu Hospital, Capital Medical University, Beijing 100053, P. R. China

^2^Department of Medical Laboratory of Beijing Children’s Hospital, Capital Medical University, Beijing 100053, P. R. China

^*^Corresponding author: Prof. Peichang Wang, Clinical Laboratory of Xuanwu Hospital, Capital Medical University, 45 Changchun Road, Beijing 100053, P. R China. Tel & Fax: +86-010-83198688; E-mail: pcw1905@163.com

1. **Supplementary Table S1-S2**

**Supplementary Table S1.** The expression changes (>2-fold) of miRs between starvation and no starvation culture

| miR | Fold change | Probe signal  (starvation) | Probe signal  (no starvation) |
| --- | --- | --- | --- |
| mmu-miR-101b-3p | 51.51 | 6.46 | 0.1 |
| mmu-miR-1188-3p | 53.66 | 6.73 | 0.1 |
| mmu-miR-1196-5p | 2.21 | 18.32 | 6.57 |
| mmu-miR-140-5p | 53.00 | 6.65 | 0.1 |
| mmu-miR-181c-5p | 56.41 | 7.08 | 0.1 |
| mmu-miR-129-1-3p | 54.48 | 6.84 | 0.1 |
| mmu-miR-1943-3p | 56.83 | 7.13 | 0.1 |
| mmu-miR-30b-5p | 58.10 | 7.29 | 0.1 |
| mmu-miR-425-5p | -52.04 | 0.1 | 6.53 |
| mmu-miR-466h-5p | -26.93 | 0.1 | 3.38 |
| mmu- miR-133a | -82.62 | 0.1 | 10.37 |
| mmu-miR-466m-5p | -48.63 | 0.1 | 6.10 |
| mmu-miR-466n-3p | -29.75 | 0.1 | 3.73 |
| mmu-miR-468-3p | -23.00 | 0.1 | 2.88 |
| mmu-miR-483-3p | -47.85 | 0.1 | 6.00 |
| mmu-miR-532-5p | -58.72 | 0.1 | 7.37 |
| mmu-miR-6368 | -98.18 | 0.1 | 12.33 |
| mmu-miR-6378 | -45.66 | 0.1 | 5.73 |
| mmu-miR-6393 | -139.27 | 0.1 | 17.49 |
| mmu-miR-299-5p | -51.40 | 0.1 | 6.45 |
| mmu-miR-669a-3-3p | -2.82 | 4.80 | 17.06 |
| mmu-miR-674-3p | -2.18 | 4.69 | 12.90 |
| mmu-miR-690 | -122.81 | 0.1 | 15.42 |
| mmu-miR-742-3p | -22.90 | 0.1 | 2.87 |
| mmu-miR-883b-3p | -47.08 | 0.1 | 5.91 |
| mmu-miR-671-5p | -2.31 | 3.51 | 6.47 |

**Supplementary Table S2. Clinical characteristics of subjects studied**

| **Sample NO.** | **Sex** | **Age(Y)** | **Years of education** | **MMSE**  **(scores)** | **MRI** |
| --- | --- | --- | --- | --- | --- |
| **Control** |  |  |  |  |  |
| **1** | M | 78 | 10 | 31 | MTLA+HA |
| **2** | F | 76 | 7 | 29 | MTLA |
| **3** | F | 73 | 10 | 32 | MTLA |
| **4** | F | 77 | 7 | 30 | MTLA |
| **5** | M | 71 | 11 | 31 | MTLA+HA |
| **6** | F | 82 | 12 | 26 | MTLA+HA |
| **AD** |  |  |  |  |  |
| **1** | F | 82 | 10 | 15 | NA |
| **2** | F | 73 | 7 | 17 | NA |
| **3** | F | 72 | 7 | 12 | NA |
| **4** | M | 81 | 12 | 10 | NA |
| **5** | F | 80 | 12 | 9 | NA |
| **6** | M | 75 | 7 | 16 | NA |

**Key:** MMSE: Mini-Mental State Examination; MRI: Magnetic Resonance Imaging; MTLA: medial temporal lobe atrophy; HA: hippocampus atrophy; NA: no atrophy in temporal lobe and hippocampus.

1. **Supplementary Figure S1-S13**


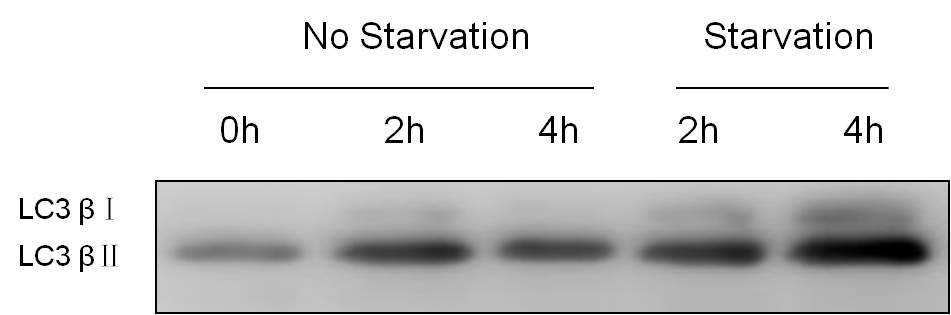


**Supplementary Figure S1a.** The protein level change of LC3βII at different time point under nutrient-deprivated culture condition. LC3βII protein levels were enhanced by nutrient starvation culture for 4 h.


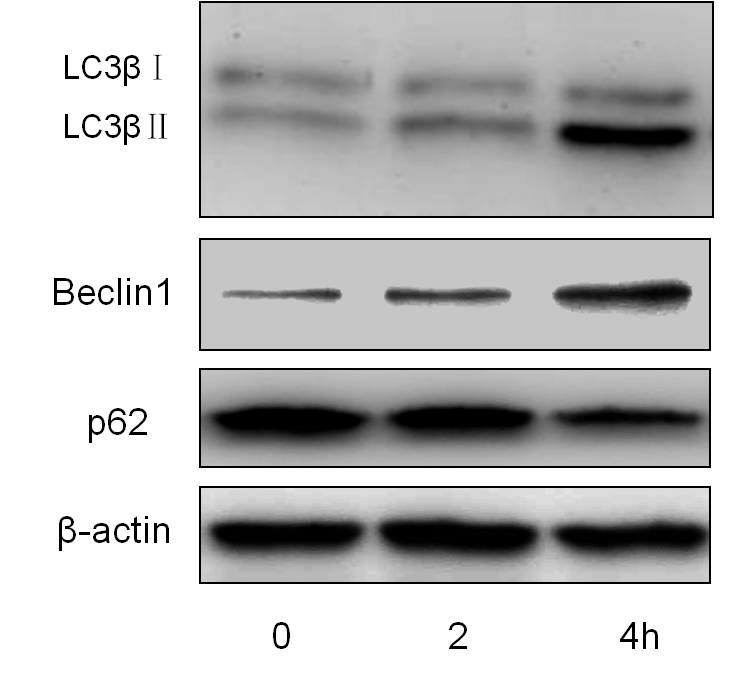


**Supplementary Figure S1b.** After 4 hours culture in EBSS autophagy was significantly enhanced in primary hippocampal neurons.

**Supplementary Figure S2.** Densitometric analysis of the data in Figure 1b. Data represent the mean ± SD of the band intensities for each protein relative to the intensities of β-actin from 3 independent experiments and were evaluated by Image J software. *: P<0.05, **: P<0.01, #: no significance.

**Supplementary Figure S3a.** Densitometric analysis of the data in Figure 2b. Data represent the mean ± SD of the band intensities for each protein relative to the intensities of β-actin from 3 independent experiments. *: P<0.05.

**Supplementary Figure S3b.** Densitometric analysis of the data in Figure 2f. Data represent the mean ± SD of the band intensities for each protein relative to the intensities of β-actin from 3 independent experiments. *: P<0.05, #: no significance.

**Supplementary Figure S3c.** Densitometric analysis of the data in Figure 2g. Data represent the mean ± SD of the band intensities for each protein relative to the intensities of β-actin from 3 independent experiments. *: P<0.05, #: no significance.

**Supplementary Figure S3d.** Densitometric analysis of the data in Figure 2h. Data represent the mean ± SD of the band intensities for each protein relative to the intensities of β-actin. n=4 per group, *: P<0.05.


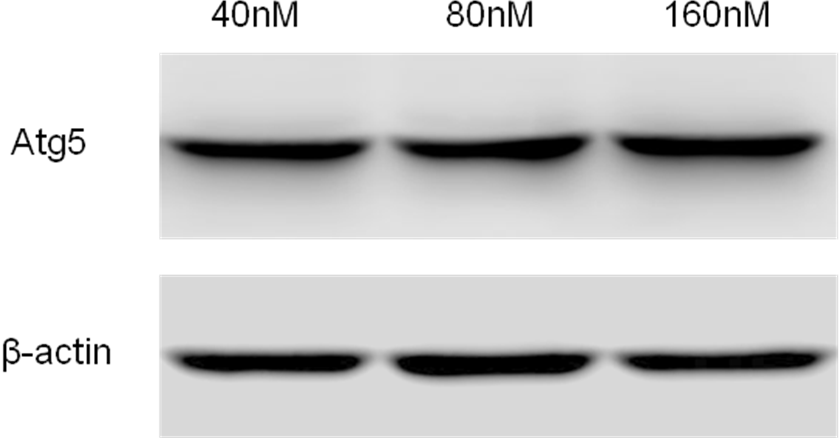


**Supplementary Figure S4a.** The Atg5 protein levels after treatment with AM299-5p in SH-SY5Y cells .There was a trend but no significant change on Atg5 expression levels after treatment with AM299-5p under different concentration.


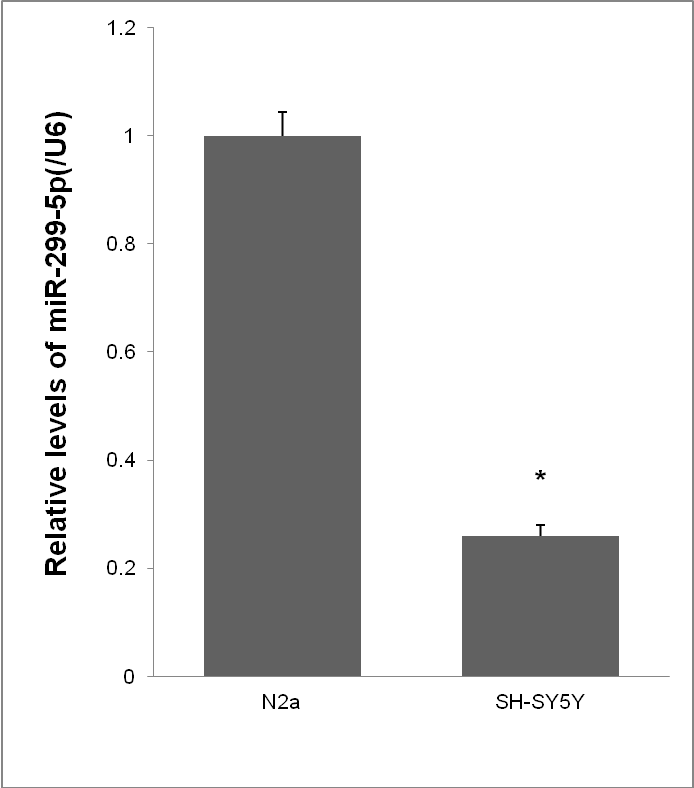


**Supplementary Figure S4b.** The relative level of miR-299-5p in SH-SY5Y cells compared with N2a cells. n=3, *: P<0.05.


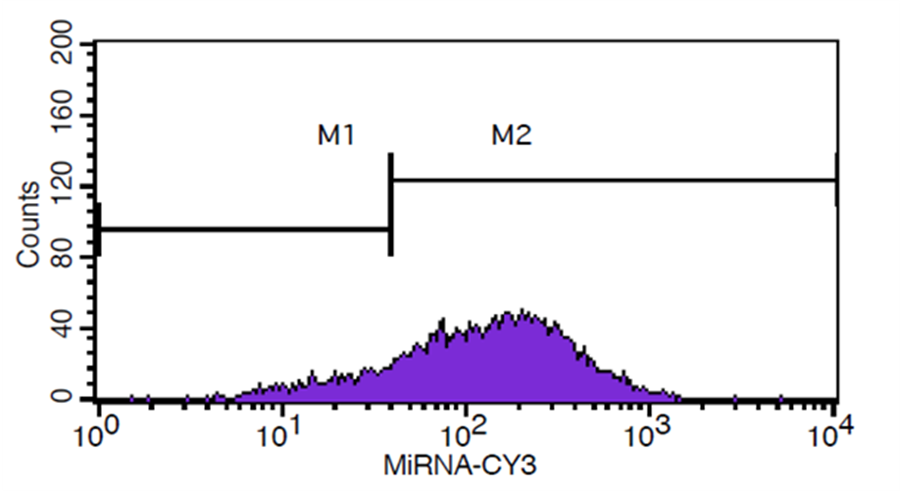


**Supplementary Figure S5.** The transfection efficiency of Ago299-5p into primary hippocampal neurons (approximately 87%).


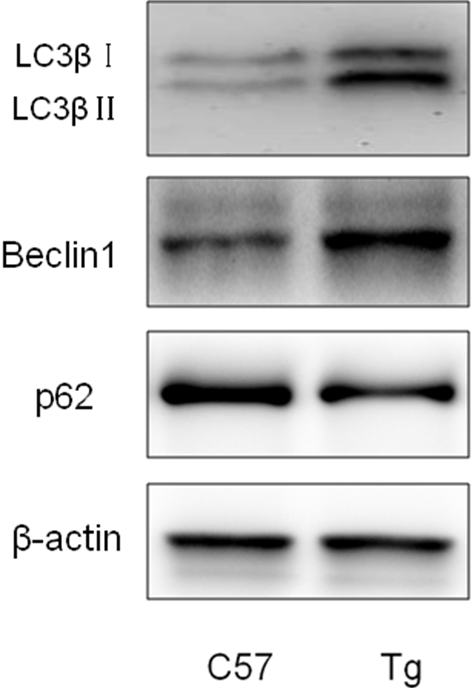


**Supplementary Figure S6.** The autophagic protein in hippocampal neurons from APPswe /PS1dE9 mice and C57 mice.

**
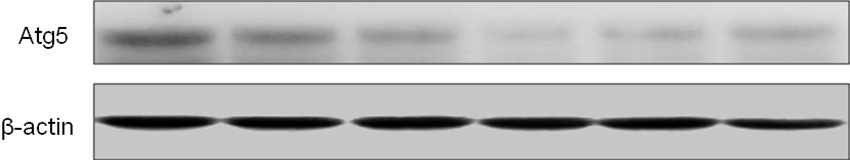
**

6

12

18

24

36

48h

**Supplementary Figure S7.** Time-dependent change of Atg5 after the transfection of Ago299-5p in primary AD mice neurons.


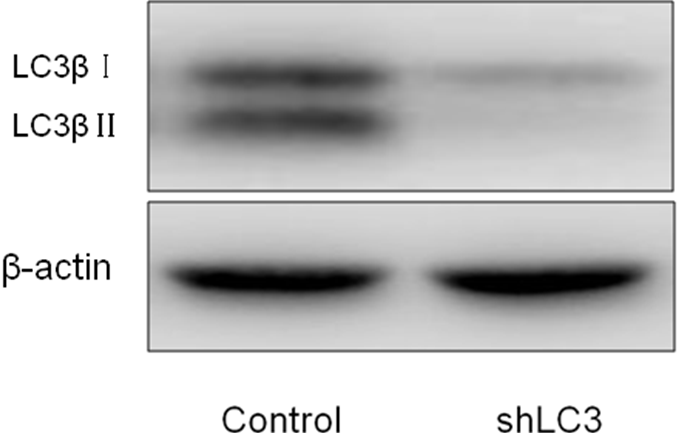


**Supplementary Figure S8.** Protein expression in primary neurons interfered by MAP LC3 shRNA Lentiviral Particles.

**Supplementary Figure S9.** Levels of apoptosis-related proteins in primary neurons 24 h after transfection.**.**

**Supplementary Figure S10a.** Densitometric analysis of the data in Figure 4c. Data represent the mean ± SD of the band intensities for each protein relative to the intensities of β-actin from 3 independent experiments. *: P<0.05, #: no significance.

**Supplementary Figure S10b.** Densitometric analysis of the data in Figure 4d. Data represent the mean ± SD of the band intensities for each protein relative to the intensities of β-actin from 3 independent experiments. *: P<0.05, #: no significance.

**Supplementary Figure S11a.** Densitometric analysis of the data in Figure 6a. Data represent the mean ± SD of the band intensities for each protein relative to the intensities of β-actin. n=5 per group, *: P<0.05, #: no significance.

**Supplementary Figure S11b.** Densitometric analysis of the data in Figure 6b. Data represent the mean ± SD of the band intensities for each protein relative to the intensities of β-actin. n=5 per group *: P<0.05, #: no significance.


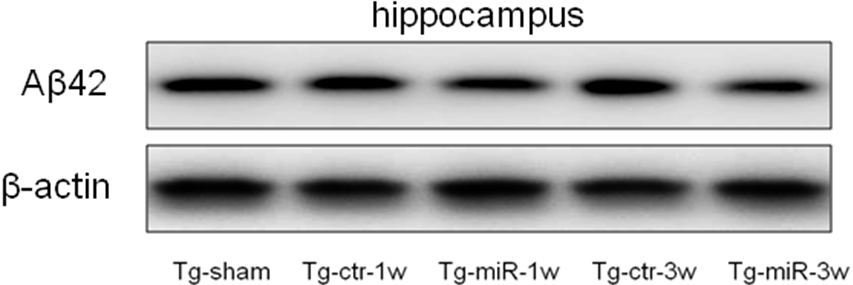


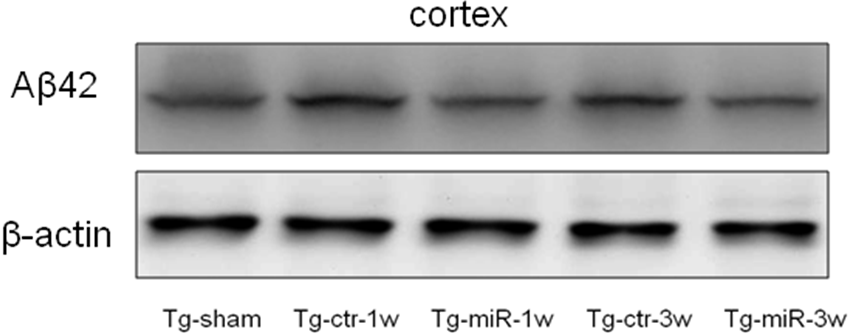


**Supplementary Figure S12a.** Western blot for Aβ42 expressions in hippocampus and cortex. There was no significant difference of Aβ42 deposited in hippocampus or cortex among five groups.


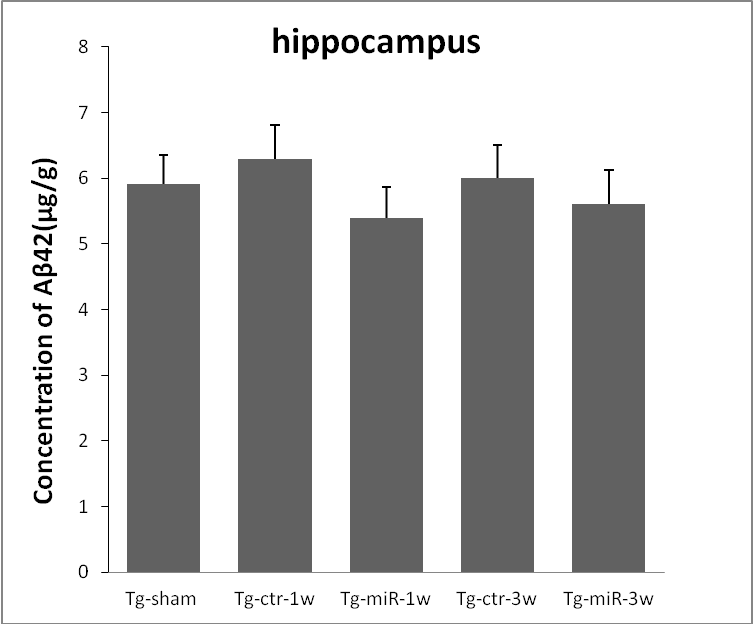

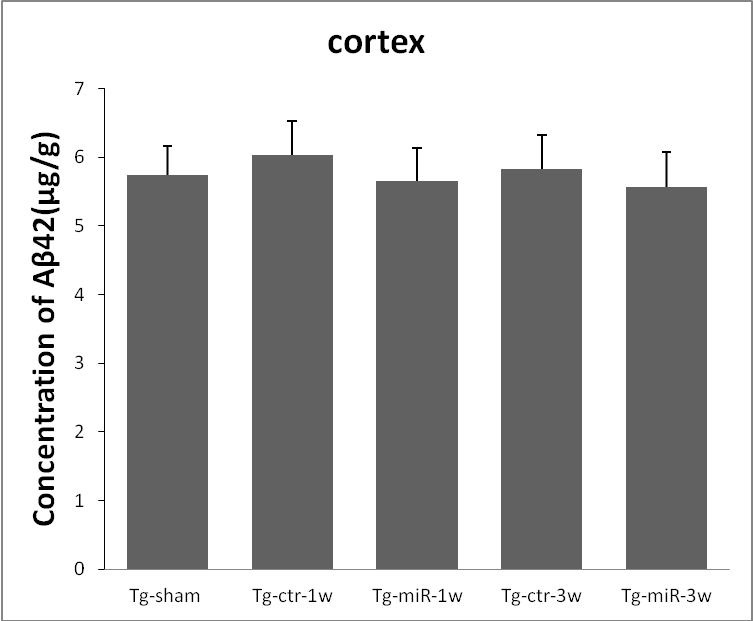


**Supplementary Figure S12b.** ELISA detection for Aβ42.AgomiR-299-5p had no effect on the total Aβ42 expression in hippocampus or cortex among five groups. n=5 per group.


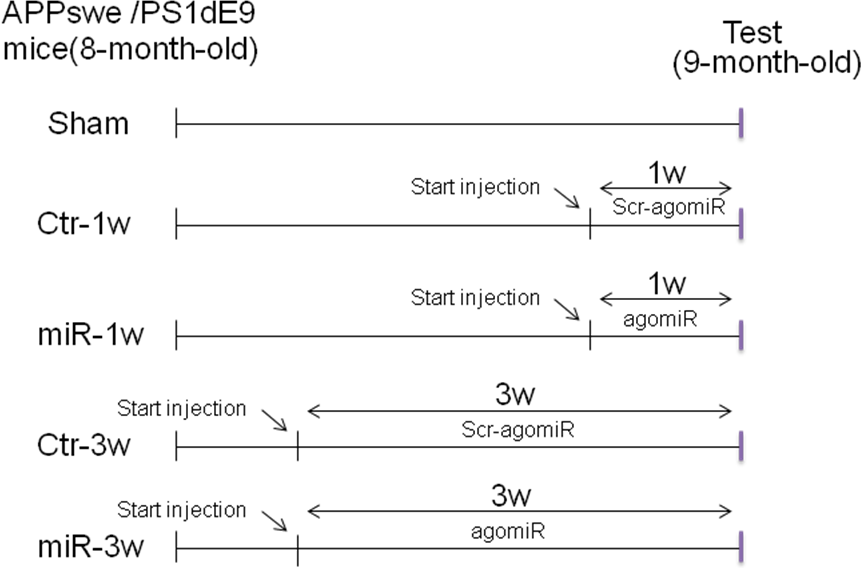


**Supplementary Figure S13.** Experimental schedules for intraventricular injection. APPswe /PS1dE9 mice were treated with agomiR-299-5p (miR) or scrambled agomiR (Ctr) for 1 or 3 weeks before behavioral test or sacrifice at 9 months of age.
